# Expert consensus on pediatric orthodontic therapies of malocclusions in children

**DOI:** 10.1038/s41368-024-00299-8

**Published:** 2024-04-16

**Authors:** Chenchen Zhou, Peipei Duan, Hong He, Jinlin Song, Min Hu, Yuehua Liu, Yan Liu, Jie Guo, Fang Jin, Yang Cao, Lingyong Jiang, Qingsong Ye, Min Zhu, Beizhan Jiang, Wenhua Ruan, Xiao Yuan, Huang Li, Rui Zou, Yulou Tian, Li Gao, Rui Shu, Jianwei Chen, Renkai Liu, Shujuan Zou, Xiaobing Li

**Affiliations:** 1 State Key Laboratory of Oral Diseases & National Center for Stomatology & National Clinical Research Center for Oral Diseases & Department of Pediatric Dentistry, West China Hospital of Stomatology, Sichuan University, Chengdu, China; 2 State Key Laboratory of Oral Diseases & National Center for Stomatology & National Clinical Research Center for Oral Diseases & Department of Orthodontics, West China Hospital of Stomatology, Sichuan University, Chengdu, China; 3 State Key Laboratory of Oral and Maxillofacial Reconstruction and Regeneration & Key Laboratory of Oral Biomedicine Ministry of Education & Hubei Key Laboratory of Stomatology & Department of Orthodontics & Center for Dentofacial Development and Sleep Medicine, School and Hospital of Stomatology, Wuhan University, Wuhan, China; 4 Chongqing Key Laboratory of Oral Diseases and Biomedical Sciences & Chongqing Municipal Key Laboratory of Oral Biomedical Engineering of Higher Education, Stomatological Hospital of Chongqing Medical University, Chongqing Medical University & College of Stomatology, Chongqing Medical University, Chongqing, China; 5 Department of Orthodontics, Hospital of Stomatology, Jilin University, Changchun, China; 6 Department of Orthodontic & Oral Biomedical Engineering Laboratory, Shanghai Stomatological Hospital, Fudan University, Shanghai, China; 7 Department of Orthodontics, Central Laboratory, Peking University School and Hospital for Stomatology & National Center for Stomatology & National Clinical Research Center for Oral Diseases & National Engineering Research Center of Oral Biomaterials and Digital Medical Devices & Beijing Key Laboratory of Digital Stomatology & Research Center of Engineering and Technology for Computerized Dentistry Ministry of Health & NMPA Key Laboratory for Dental Materials & National Engineering Research Center of Oral Biomaterials and Digital Medical Devices, Beijing, China; 8 Department of Orthodontics, School and Hospital of Stomatology, College of Medicine, Shandong University & Shandong Key Laboratory of Oral Tissue Regeneration & Shandong Engineering Laboratory for Dental Materials and Oral Tissue Regeneration & Shandong Provincial Clinical Research Center for Oral Diseases, Shandong University, Jinan, China; 9 State Key Laboratory of Oral & Maxillofacial Reconstruction and Regeneration, National Clinical Research Center for Oral Diseases, Shaanxi Clinical Research Center for Oral Diseases, Department of Orthodontics, School of Stomatology, Air Force Medical University, Xi’an, China; 10 Hospital of Stomatology, Guangdong Provincial Key Laboratory of Stomatology, Guanghua School of Stomatology, Sun Yat-Sen University, Guangzhou, China; 11 Center of Craniofacial Orthodontics, Department of Oral and Cranio-Maxillofacial Surgery, Shanghai Ninth People’s Hospital, Shanghai Jiao Tong University School of Medicine & College of Stomatology, Shanghai Jiao Tong University & National Center for Stomatology, National Clinical Research Center for Oral Diseases, Shanghai Key Laboratory of Stomatology, Shanghai Research Institute of Stomatology, Shanghai, China; 12 Center of Regenerative Medicine, Department of Stomatology, Renmin Hospital of Wuhan University, Wuhan, China; 13 Department of Oral and Cranio-maxillofacial Surgery, Shanghai Ninth People’s Hospital, Shanghai Jiao Tong University School of Medicine & National Clinical Research Center for Oral Diseases & Shanghai Key Laboratory of Stomatology & Shanghai Research Institute of Stomatology, Shanghai, China; 14 Department of Pediatric Dentistry, School and Hospital of Stomatology, Tongji University & Shanghai Engineering Research Center of Tooth Restoration and Regeneration, Shanghai, China; 15 Department of Stomatology, The Children’s Hospital, Zhejiang University School of Medicine & National Clinic Research Center for Child Health, Hangzhou, China; 16 Department of Orthodontics, The Affiliated Hospital of & School of Stomatology, Qingdao University, Qingdao, China; 17 Department of Orthodontics, Nanjing Stomatological Hospital, Medical School of Nanjing University, Nanjing, China; 18 Key Laboratory of Shaanxi Province for Craniofacial Precision Medicine Research, Clinical Research Center of Shaanxi Province for Dental and Maxillofacial Diseases & College of Stomatology, Xi’an Jiaotong University & Department of Orthodontics, Xi’an Jiaotong University, Xi’an, China; 19 Department of Orthodontics, School and Hospital of Stomatology, China Medical University & Liaoning Provincial Key Laboratory of Oral Diseases, Shenyang, China; 20 Department of Pediatric Dentistry, The First Affiliated Hospital of Zhengzhou University, Zhengzhou, China

**Keywords:** Dental diseases, Craniofacial orthodontics

## Abstract

Malocclusion, identified by the World Health Organization (WHO) as one of three major oral diseases, profoundly impacts the dental-maxillofacial functions, facial esthetics, and long-term development of ~260 million children in China. Beyond its physical manifestations, malocclusion also significantly influences the psycho-social well-being of these children. Timely intervention in malocclusion can foster an environment conducive to dental-maxillofacial development and substantially decrease the incidence of malocclusion or reduce the severity and complexity of malocclusion in the permanent dentition, by mitigating the negative impact of abnormal environmental influences on the growth. Early orthodontic treatment encompasses accurate identification and treatment of dental and maxillofacial morphological and functional abnormalities during various stages of dental-maxillofacial development, ranging from fetal stages to the early permanent dentition phase. From an economic and societal standpoint, the urgency for effective early orthodontic treatments for malocclusions in childhood cannot be overstated, underlining its profound practical and social importance. This consensus paper discusses the characteristics and the detrimental effects of malocclusion in children, emphasizing critical need for early treatment. It elaborates on corresponding core principles and fundamental approaches in early orthodontics, proposing comprehensive guidance for preventive and interceptive orthodontic treatment, serving as a reference for clinicians engaged in early orthodontic treatment.

## Introduction

Malocclusion ranks among the top three oral diseases as identified by the World Health Organization. This condition profoundly impacts the dental-maxillofacial functions, facial esthetics, and the growth and development of ~260 million children in China.^1,2^ Beyond its physical manifestations, malocclusion also significantly influences the psycho-social well-being of these children. The emergence of malocclusion is influenced by both genetic and environmental factors. Timely intervention in malocclusion can foster a conducive environment for dental-maxillofacial development, thereby mitigating the negative impact of abnormal environmental and genetic influences on dental-maxillofacial growth. Such early interventions can substantially decrease the incidence of malocclusion in children, thereby enhancing their overall physical and mental health. From an economic and societal standpoint, the urgency for effective early orthodontic treatments for malocclusion in children cannot be overstated, underlining its profound practical and social importance. This consensus paper outlines the detrimental effects of malocclusion, underscores the critical need for early treatment, and introduces core principles and fundamental approaches. It proposes guidance for preventive and interceptive orthodontic interventions across various stages of dental-maxillofacial growth and development, from fetal stages to the early permanent dentition phase. This paper emphasizes the necessity of a systematic approach to promote and standardize early orthodontic treatment, underscoring its significance in improving children’s health outcomes. Early intervention for malocclusion in childhood involves timely identification and treatment of dental-maxillofacial morphological and functional abnormalities during various stages of development. Through effective and rational intervention, this approach aims to eliminate oral and systemic environmental factors that adversely affect dental-maxillofacial development. It also seeks to reduce the severity and complexity of malocclusion, thereby achieving harmonious and esthetically pleasing dental-maxillofacial structures and functions with greater efficiency. This consensus paper will elaborate on the characteristics of childhood malocclusion and the corresponding principles of preventive treatment. It proposes guidelines and fundamental principles for early intervention, serving as a reference for clinicians engaged in early orthodontic treatment.

## Definition and risk of malocclusion

Malocclusion is a multi-factorial condition in children characterized by abnormal growth and development, influenced by genetic and environmental factors including dental and pulp diseases, trauma, oral habits, dental replacement disorders, among others. This condition leads to misalignment of teeth, abnormalities in the relationship between the upper and lower dental arches, morphological and positional abnormalities in jaw size, and facial deformities, among other manifestations.

### Oral functions

Malocclusion seriously affects children’s oral functions, including mastication, swallowing, speech and temporomandibular joint functions. Occlusion characterized by deep overbite, open bite, or occlusal interference results in deviations in the range and trajectory of mandibular movements during opening, protrusion, and lateral movements. This may induce perioral muscle fatigue and/or degeneration, subsequently affecting the temporomandibular joint function and potentially resulting in organic pathology.^3–11^ Atypical tongue posture and swallowing patterns often contribute to the onset and progression of malocclusion, acting as predisposing factors rather than merely being correlated with it.^12–18^ Moreover, malocclusion can impact articulation, as exemplified by individuals presenting skeletal Class III malocclusion who may experience speech impairments.^19–23^ Malocclusion, characterized by misalignment, along with abnormalities in the size, shape, and number of teeth can reduce the functional contact area of the maxillary and mandibular teeth and result in reduced masticatory efficiency.^24–26^

### Oral health

The accumulation of food debris, plaque, and the subsequent increased formation in calculus resulting from dental crowding and misalignment poses challenges in effective oral hygiene maintenance. Such conditions can give rise to caries, gingival periodontal diseases, and periapical disease, among other oral health concerns.^27–29^ In other cases, occlusal trauma caused by premature contact has the potential to exacerbate periodontal disorders,^30,31^ while the anterior protrusion of upper incisors heightens the susceptibility to dental trauma.^32–39^

### Dental-maxillofacial growth

In the context of children’s growth and development, malocclusion affects dental and maxillofacial development, impacting both soft and hard tissues. For instance, an anterior crossbite compounded by a potential abnormally anterior positioning of the mandible may impede the sagittal growth of the maxilla and stimulate excessive mandibular growth, consequently disrupting the balance of the sagittal relationship between the maxilla and mandible. In the other case, excessive narrowing of the upper dental arch or upright upper anterior teeth may restrict mandibular protrusion, impeding its development. Additionally, unilateral posterior crossbite or occlusal interferences will result in asymmetric facial development.^40,41^

### Physical and mental health

Malocclusion is correlated with general development of children’s whole body. Impaired chewing function resulting from malocclusion may lead to a reduction in nutrition intake.^42^ Mandibular retrusion is one of the contributing factors to obstructive sleep apnea, also called obstructive sleep apnea hypopnea syndrome, whose associated consequences include sleep disturbances, neurocognitive issues, and attention deficits.^43^ Apart from physical health, malocclusion, especially those leading to a poor facial appearance, affects children’s psychological health, forming low self-esteem. Increased overjet and deep bite, space between anterior teeth, and extremely misaligned teeth are potential reasons for children to be teased,^44–46^ causing the affected person isolated from the social activities and difficult to interact with others.

## Mechanism of the occurrence of malocclusion

The etiology of malocclusion involves multi-factorial influences. One influential factor can cause different types of malocclusion, while a single malocclusion can be attributed to different influential factors acting in a complexity mode.^47^ Therefore, it is important to analyze the etiological mechanism during the diagnosis and treatment of malocclusion. More importantly, we need to provide proper solutions to eliminate the influential factor in early orthodontic interventions.^48^

Based on current understanding, it is believed that the development of children’s teeth and maxillofacial complex is affected by various factors, including genetics, environment, diseases, and injury.^49^ Genetic factors control craniofacial morphology, structure and size, whereas environmental factors exert a greater influence on the growth and development of alveolar and jawbones.^50–53^ In the genetic context, environmental factors exacerbate the malocclusion manifestation.^54^ At the systemic level, craniofacial congenital malformations and genetic diseases cause abnormal craniofacial morphology. Main localized environmental factors, such as abnormal respiratory and swallowing functions, inadequate mastication, poor oral habits, and oral health, affecting dental and maxillofacial growth and development at the local level.

### Genetic factors

The genetic factors of malocclusion are closely correlated with racial evolution and personal growth. With the human evolution, dietary patterns have changed, leading to a gradual degeneration of chewing muscles, jawbone, and teeth. Given the inconsistent degree of jawbone and teeth degeneration, the lack of coordination between the mass of teeth and bone results in tooth crowding and malocclusion. Malocclusion, when inherited from parents. poses challenges to correction due to genetic factors.^55^ Therefore, clear and early diagnosis and treatment planning are demanded, aiming to compensate for the imbalanced craniofacial growth. Early orthodontic treatment should involve selecting appropriate methods and establishing reasonable staged treatment goals. It is important to ensure an extended period of maintenance and adhere to a long-term follow-up treatment regime after early orthodontic treatment.

### Disease factors

Throughout the growth and development process from embryonic period to childhood, various diseases can lead to the occurrence of malocclusion Maternal malnutrition or diseases during pregnancy can cause fetal dental and maxillofacial dysplasia or developmental abnormalities.^56^ Some acute and chronic diseases in childhood can affect teeth, jaw, and face growth, as well as total health.^57^ For example, vitamin D deficiency causes calcium and phosphorus metabolism disorders, hypothyroidism, pituitary gigantism and other diseases, which would lead to malformation of jawbone and dental arch development,^58–60^_._

### Localized environmental factors

Malocclusion is often caused by local disorders during tooth replacement in children. Early loss or retention of deciduous teeth can lead to succeeding permanent teeth eruption disorders and dislocation. Another common local environmental factor is deleterious oral habits. The unilateral chewing habit may result in posterior teeth crossbite on the chewing side, a shift in the midline of the upper and lower arches toward the chewing side, and ultimately, facial asymmetry.^40,41^ Non-nutritive sucking (NNS) such as finger-sucking, lead to anterior open bite and dental-arch narrowness.^61–65^ Thrusting the tongue and licking the anterior teeth during eruption may also contribute to localized anterior open bite. Additionally, biting the upper lip causes mandibular protrusion and anterior crossbite.^66–69^

## Main contents of early orthodontic treatment

Malocclusion, a variation from normal occlusion, is not classified as a disease but a developmental condition that often manifests during the transition to permanent dentition. Early orthodontic treatment of malocclusion plays a crucial role in managing oral health and functions in children. The goal is to create a conducive environment for the dental and maxillofacial development of children based on the growth potential of the dentition and jaw. This involves targeted interventions aimed at the prevention, guidance, and interception of malocclusion during its early developmental stages, where various orthodontic techniques and methods are applied with the aim of mitigating the severity and complexity of malocclusion.^70–72^

Early correction of malformation in children mainly includes:

*Management of oral functionality development:* oral chewing, swallowing, breathing and speech, and correction of bad oral habits.

*Management of occlusal development:* management of dental developmental abnormalities (abnormal tooth eruption, number, or morphology), management of tooth replacement abnormalities (space management), orthopedic treatment of alveolar bone developmental abnormalities, and early correction of dental malocclusion that affects oral health, function and growth and development of children.

*Management of maxillofacial growth and development:* early treatment of abnormal muscle functionality and functional malocclusion such as occlusal interferences affecting craniofacial development, comprehensive treatment of craniofacial growth and development abnormalities caused by abnormal oral functionality, orthopedic treatment of mild, moderate, and severe disharmonies of jaws in children, control of hereditary craniofacial morphological abnormalities.

*Management of general health and development:* including children’s nutritional health maintaining, early treatment of children’s respiratory diseases, correction of children’s abnormal body and head posture, prevention and treatment of children’s craniofacial trauma (condylar and jaw fractures).^73–79^

## Principals of early orthodontic treatment

**Fig. 1:**
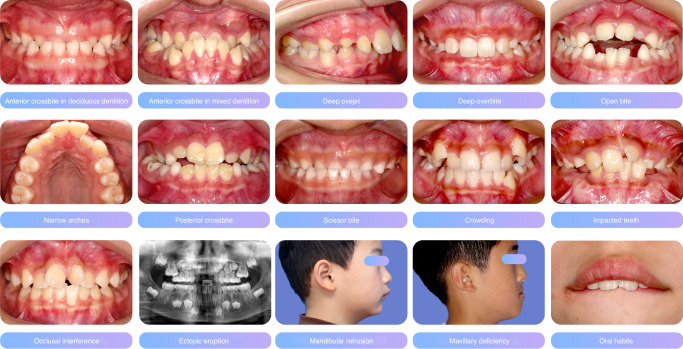
Manifestations of malocclusion that require early orthodontic treatment

Early orthodontic treatment leverages the dental and jaw growth potentials in children to prevent and interrupt the development of malocclusion, achieving the staging treatment goals^73^ (Fig. 1).

The treatment purpose of early intervention of malocclusion in children must be clearly defined, with an emphasis on accurate diagnosis and reasonable treatment plan as fundamental aspects for early orthodontic treatments. It’s crucial for early orthodontic treatment to be both effective and efficient, with clinicians balancing the cost and benefits involved. Modern early intervention methods for malocclusion in children combine appliance treatments with training of oral and perioral muscle functions. The available orthodontic appliances are primarily categorized into removable and fixed appliances, along with segmental fixed multi-bracket braces and clear aligners. Their functions cover palatal expanders, functional regulators, space maintainers, and oral muscle trainers. The integrated method addresses both structural and functional aspects of malocclusion, reflecting advancements in pediatric orthodontic theory.

Early orthodontic treatment of malocclusion in children also carries certain limitations and risks, requiring careful consideration in clinical decision-making. Due to the active period of dental and maxillofacial growth and development, manifestations of malocclusion in children may not be fully expressed, with some skeletal deformities or growth patterns potentially remaining stationary until growth is completed. In addition, accurately predicting growth patterns and amounts still presents some difficulties, particularly in forecasting the prognosis of genetic and/or skeletal issues, hence caution is advised regarding prolonged treatments, sequential tooth extraction, and other irreversible interventions. Simultaneously, it is imperative to acknowledge the limitations inherent in dentofacial growth modification. Our current repertoire of techniques for restraining jaw growth or fostering mandibular ramus growth remains relatively restricted. In instances where evidence is insufficient to substantiate early intervention, a conservative stance is warranted.

Specific challenges also exist during the process of early orthodontic treatment. For instance, primary teeth may exhibit shorter crowns and greater occlusal tapering, posing challenges for the bonding or retaining of orthodontic appliances. Children’s immature mindset may also impede compliance with treatment, resulting in insufficient wearing time of removable appliances and poor orthodontic effects. Poor oral hygiene and habits can lead to relapse of malocclusions or complications, such as dental caries and periodontal issues. Moreover, children’s tooth roots and alveolar bone are currently in a developmental state. It is essential for clinicians to closely monitor them and be vigilant in preventing potential treatment complications.

During the transition from primary to mixed dentition, children may experience conditions such as mild incisor crowding of less than 2 mm, maxillary midline diastema of less than 2 mm, divergence of lateral and central incisor crowns, molar half cuspid distal relationship, and mild deep bite, owing to factors like incisor liability and Leeway Space, which essentially represent asynchronous adjustments between bone and tooth mass. These occurrences are typically normal and may resolve spontaneously to establish a normal occlusion, requiring no treatment.^80^ Unless requested by the patient for temporary esthetics, minimally invasive approaches should be favored during this period.^81–84^

## Clinical examination and diagnosis of malocclusion in children

**Fig. 2:**
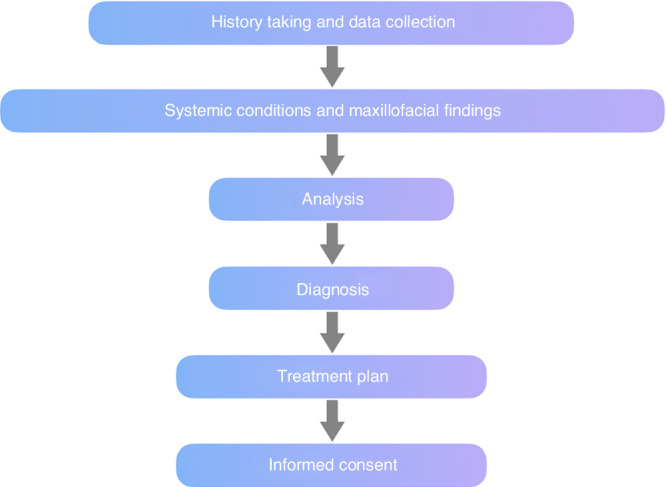
Progress of clinical examination and diagnosis of malocclusion in children

The clinical examination and diagnosis of early orthodontic treatment comprehend oral health and function, dental and maxillofacial morphology and structures, and general health development. Special attention should be given to evaluating occlusal development and relationships, as well as upper respiratory tract health^85^ (Fig. 2).

*Oral occlusal development examination:* tooth age, tooth eruption, occlusal relationship, and oral health examination. The number of deciduous permanent teeth and mandibular symmetry are examined by panoramic radiography.

*Facial morphological function examination:* facial symmetry, facial lateral appearance and growth type, breathing, swallowing, facial muscle function, jaw opening and closing movement.

*Craniofacial morphological and structural examination:* cephalometric analysis, cervical spine development analysis, tooth eruption and morphological abnormalities exam by cone-beam computed tomography (CBCT).

*Oral Habits:* through interviews and chairside observations, assess whether the child has oral habits such as lip biting, finger sucking, or tongue thrusting.

*Children’s growth and development potentials:* analysis of the second, third, and fourth cervical vertebrae morphology using lateral cephalometric films to determine the growth and development period of children.^86–88^

*Upper respiratory tract examination in children:* using lateral head films, check for hypertrophic adenoids and tonsils (whether they affect the patency of the upper respiratory tract) and, if necessary, consult an otolaryngologist.

*General development and health checkup:* For children with obvious systemic developmental abnormalities (growth hormone, shape abnormalities, craniofacial morphology, etc.), a consultation with a pediatrician is requested before starting early correction and treatment of systemic health developmental abnormalities.

## Principals of malocclusion prevention in children

The general principles of child malocclusion prevention include:

*Early orthodontic treatment in children begins with eugenics:* The fetal period is an important stage of dental and maxillofacial development. Attention at this stage can avoid the occurrence of many congenital genetic diseases.^89–95^

*Good oral hygiene habits:* Maintaining good oral hygiene habits is effective in ensuring healthy and balanced oral soft and hard tissues in functions. Intact, healthy and functionally balanced oral soft and hard tissues are prerequisites for good child facial development.

*Early detection and treatment of oral diseases:* including caries, periapical disease, mucosal disease, abnormal tethering attachment soft and hard tissue diseases that affect oral function.

*Good oral function habits:* respiratory, swallowing, language and other neuromuscular habits.

*Early detection and correction of abnormal oral functional habits:* including abnormal breathing, swallowing, chewing, tongue thrusting, lip licking, finger sucking, cheek sucking, abnormal head and neck posture, abnormal full-body posture, etc.

## Clinical practice of early orthodontic treatment in different growth stages

### Orthodontic treatment in primary dentition (2.5/3–6 years of age)

*Oral hygiene habits:* Cultivating optimal oral hygiene practices should start with oral cleaning from birth. Parents should prioritize maintaining a proper feeding posture during this period, advocate breastfeeding, avoid bad bottle feeding posture, pay attention to chewing function training, and avoid too soft food.^96–98^

*Oral functional habits:* Parents should be vigilant in the timely detection and correction of various detrimental oral habits that may emerge after the oral appetite period, such as the continued use of the pacifier and thumb or finger sucking.^61,99,100^ It is important to introduce proper oral functions, such as lip closure training and tongue exercises. Additionally, preventing trauma to the deciduous teeth is essential.^101–103^

*Crossbite correction:* Crossbite in primary dentition should be corrected with orthodontic removable appliances, such as a removable appliance with recurved spring and a posterior bite plane, a bonded lower anterior acrylic slide plane for anterior crossbite, and removable or bonded Schwartz expanders with occlusal coverage for posterior crossbite. Chin caps can correct the bad habits of mandibular advancement during the primary dentition. For children with functional protruding mandible with tonsillar hypertrophy, otolaryngology should be consulted while correcting anterior crossbite, and the hypertrophied tonsils should be removed promptly if necessary to avoid secondary mandibular protrusion. For children with hereditary maxillary hypoplasia, maxillary expansion with anterior traction or Frankel III (functional regulator III, FR III) is feasible for early treatment to promote maxillary development.

*Other dental malocclusion:* During the primary dentition period, malocclusion, such as severe anterior deep overbite, significant arch narrowing, anterior protrusion of upper incisors, and posterior crossbite, combined with abnormal oral functions causing the child’s palatal mucosa injuries should be treated promptly. A common condition is anterior deep overbite causing the trauma of the palatal mucosa.

Periodically detection and correction of all kinds of bad oral habits are needed. The correction of habits such as abnormal sucking, biting, and incomplete lip closure should begin after 3.5 years of age.

*Skeletal disorders:* Children with abnormal facial growth patterns in the primary dentition, such as anterior maxillary protrusion, clockwise rotation of the lower jaw, and a noticeable acute mandibular angle, may be temporarily unable to undergo systematic orthopedic treatment. Instead, primary adjunctive therapy could involve oral functional training.

*Space maintenance:* In the case of premature loss of primary deciduous molars and mandibular primary canines, space maintenance is required104.

*Other diseases:* Clinicians should promptly detect and treat ear, nose, and throat (ENT) diseases, and other respiratory diseases, and treat oral soft and hard tissue diseases that may cause changes in the functional environment of the oral cavity.

### Orthodontic treatment in mixed dentition (6–12 years of age)

Early orthodontic treatment in the mixed dentition should primarily focus on differentially diagnosing the physiological malocclusions of mixed dentition. Temporary malocclusion in mixed dentition should be observed without urgent orthodontic treatment:

1. maxillary midline diastema (<2 mm): mostly caused by the lateral incisor germ pressing the root of the central incisors, which can self-correct with the eruption of the lateral incisor.
2. distal tipping lateral incisors: mostly caused by the canine’s germ pressing the root of the lateral incisors.
3. mild to moderated anterior deep bite, characterized by minimal lingual inclination of upper incisors: can self-adjust with the growth of the mandible and the increase of alveolar bone height and molar crown height after the completion of tooth replacement.
4. mild crowding of incisors (incisor liability): typically resolved by the labial eruption of anterior teeth, utilization of the primate space, and natural growth of the arch width.
5. mild distal relationship of first permanent molars: can be adjusted with the replacement of the primary molars by premolars, in which upper and lower first permanent molars drift mesially differently (using Leeway Space) and differential growth of the maxilla and mandible (mandibular growth retardation).

The basic elements of early orthodontic treatment in children with mixed dentition include:

*Anterior Cross Bite:* Timely treatment of dental and functional anterior crossbite is crucial, which should be detected and addressed promptly. The main purpose of anterior crossbite correction is to interrupt functional mandibular advancement, preventing the progression of a dental problem to a skeletal problem. For skeletal Class III malocclusions caused by genetic factors, noticeable maxillary and mandibular discrepancy during this period. For children with predominantly genetic maxillary underdevelopment, early treatment with maxillary expansion with anterior traction or FR III orthodontic appliances can promote maxillary development,^104–106^ while efforts should be made to control mandibular overgrowth or allow slight compensation through mandibular rotation. In obviously severe hereditary skeletal anterior crossbite, early clinical controls of mandibular growth could be facilitated, but clinical efficacy is uncertain.

Tonsillar hypertrophy is a risk factor for the development of skeletal anterior crossbite in children. Children with anterior crossbite and oversized tonsils should be promptly referred to an ENT specialist for evaluation. If necessary, tonsillectomy should be considered along with functional orthodontic treatment to control mandibular advancement.

*Anterior deep bite and deep overjet with labial inclination:* Treatment involves uprighting proclined upper incisors, lingually tipping lower incisors, coordinating of upper and lower arch width, and timely correction of anterior deep overjet. This treatment should be complemented with lip competency improvement to alleviate the adverse effects of weak perioral muscle function on exacerbating deep overjet. Additionally, correct oral sucking habits if necessary. Early and timely bite opening can improve vertical growth of the jaws and increase the height of the lower third of the face, changing the horizontal growth pattern of the face.^107^ Early correction of deep overjet with labial tipped of the upper incisors can prevent incisor fractures in children.

*Facial protruding profiles in children:* For patients with Skeletal Class II malocclusion with the functional mandibular recession, the upper and lower arch widths are checked for their coordination, and the arch can be expanded early to release functional mandibular recession due to the narrow upper arch.

Functional orthodontic appliances of mandibular advancement are the main orthodontic appliances used during this period, and the timing of orthodontic treatment is during the pre- or during the peak of the pubertal growth spurt of the child.^108^

For skeletal maxillary protrusion, the growth of the protrusive maxilla should be suppressed as much as possible during the growth and functional appliances facilitated with extra-oral headgear should be used, and treatment time should start before the peak of pubertal growth.

*Early orthodontic treatment of dental developmental abnormalities:* Early detection and orthodontic treatments can alleviate occlusal interference and tooth transposition caused by abnormal tooth eruption, while also restoring chewing function and esthetic appearance of the front teeth.^109–118^ The early orthodontic tract aid eruption of the buried teeth, blocked teeth and curved root teeth will not cease the root development of the unerupted permanent teeth and cause the apical resorption, and even have the effect of reducing the severity of root curvature of curved teeth, but the biological light force (30–60 g) required.^119,120^

*Timely space management:* Timely space management is crucial in cases of premature loss of primary teeth. Except the premature loss of upper incisors, it is necessary to prevent space loss. This is especially important when the anticipated eruption of permanent successors is more than 6 months. Special attention should be paid to maintaining space after the premature loss of the upper and lower second deciduous molars and lower deciduous canines.^114,121–124^ For patients with already closed edentulous space, procedures to regain space may be necessary. This facilitates the normal replacement of deciduous and permanent teeth.

*Early maxillary expansion:* Patients should undergo early orthodontic treatment for insufficient arch widths, abnormal arch length, uncoordinated upper and lower arch forms, as well as asymmetric left and right arch forms. Maxillary bony expansion should be performed before the mid-palatal suture is closed, and the best time for maxillary expansion is from 7 to 10 years old, in which time the bone effects of early expansion is better.^116,125–129^

*Deleterious oral habits:* In mixed dentition, early identification and intervention should be performed for all kinds of bad oral habits, including mouth breathing, atypical swallowing, tongue thrusting, functional mandibular advancement, NNS (thumb, finger, cheek, or a similarly shaped object), lip biting, soft diet, bottle-feeding incompetent lip closure, unilateral chewing habits, etc.^98,130–132^ Cooperating with early orthodontic treatment and actively carrying out functional training of maxillofacial muscles should be conducted.^133–138^ Orofacial myofunctional therapy (OMT) targets oral and oropharyngeal structures, aiming to improve muscle tone, endurance, and coordinated movements in the pharynx and surrounding areas. Clinically, OMT has shown positive outcomes in enhancing swallowing function, correcting tongue posture and muscle dysfunction, and reducing the likelihood of relapse from previous orthodontic treatments.^139,140^ At this stage, if a child is unable to self-correct bad habits after reminder and reward therapy, some orthodontic appliances can be used as habit breakers for auxiliary intervention. For instance, vestibular shields can be used to block habitual mouth breathing and for lip muscle training, frog-mouth appliances can correct immature swallowing patterns, and palatal bar or tongue crib can prevent tongue thrusting and NNS. Some prefabricated myofunctional appliances made of silicone materials can be used simultaneously for occlusal guidance, habit cessation, and muscle function training.^141–147^

**Fig. 3:**
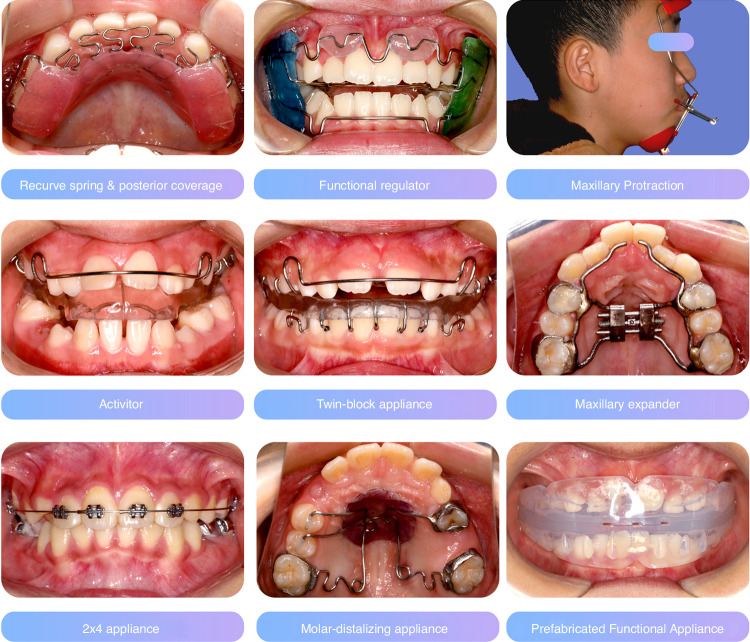
Orthodontic techniques commonly used in early orthodontic treatment

*Other diseases:* It is needed to have timely detection and treatment of otolaryngological diseases and other respiratory diseases, and oral soft and hard tissue diseases that may cause changes in the functional and mechanical environment of the oral cavity in mixed dentition^148^ (Fig. 3).

### Orthodontic treatment in early permanent dentition (after 12 years of age)

After the completion of the replacement of succeeding permanent teeth, the occlusal growth enters the early stages of permanent dentition. The second permanent molar is still to erupt, and the child is in the late stages of growth spurt of puberty. At the beginning of the permanent dentition, the speed of jaw growth and development slows down, but occlusion is not yet fully established, and growth modifications remain (especially in males).

Orthodontic treatment during this period still requires paying an attention to maintaining oral hygiene, good oral functions, and thorough correction of various oral malfunctions. Adjunctive oral muscle function training would help to stabilize the results of comprehensive orthodontic treatment of malocclusion.

*Functional orthodontic treatment for anterior crossbite:* Orthodontic functional therapy, such as FR III, may be beneficial in treating mild-to-moderate skeletal Class III malocclusions. It is recommended to track growth and development and conduct regular follow-up reviews. Due to the unpredictability of mandibular growth and development (especially in males), comprehensive orthodontic camouflage treatment in early permanent dentition should be postponed. Tooth exaction in the treatment plan for skeletal class III malocclusion should be approached with great caution to avoid complicating future orthodontic-orthognathic surgery to correct the jaw discrepancies in adulthood. For children with severe high-angle skeletal Class III malocclusion and a family history, combined orthodontic-orthognathic surgery is the appropriate treatment, eliminating the need for early intervention. Patients with maxillary hypoplasia of Class III malocclusion may benefit from anterior traction with palatal expansion or FR III orthodontic appliances in the early permanent dentition.

*Orthodontic treatment of facial convex profile in early permanent dentition:* At this stage, the mandible remains some growth potential (especially in males). For a retruded mandible, orthopedic treatment can still be performed firstly to address the sagittal discrepancy between maxilla and mandible. Note: For Class II malocclusion with maxillary width deficiency, a rapid expansion appliance to increase the maxillary arch base should be applied prior to the start of mandibular advancement or using a functional appliance with a maxillary expansion crew. Mild to moderate maxillary hypoplasia with an anteriorly protruding facial profile usually requires extraction to camouflage the skeletal protrusion. For severe skeletal maxillary protrusion, a combined orthodontic-orthognathic treatment should be chosen to correct the facial protrusion.

*Orthodontic treatment of skeletal narrow upper arch:* Due to the large variability of timing of the palatal suture closure, it is also possible to open the palatal suture during the early permanent dentition to correct skeletal width discrepancy. Most of the clinical methods are involved in fixed palatal rapid expansion and mini implant-assisted rapid palatal expansion.^149^

*Orthodontic treatment of crowding:* The appropriate orthodontic method is selected based on the analysis of tooth-sizes and alveolar bone volume. Clinical methods of space obtaining include tooth extraction, molar distalization, arch expansion, and use of Leeway Space. The amount of space gained by arch expansion in the early permanent dentition is limited and should be used with cautions. In patients of mild to moderate crowding with straight profile, non-extraction orthodontic treatment can be chosen to correct the crowding. In severe crowding, extraction should be chosen to align the teeth to maintain the normal profile.^150,151^

## Clinical management of early orthodontic treatment

Children are in the immature stage of physiological and psychological development, early orthodontic treatment is different from adult orthodontic treatment in the aspects of the selection of orthodontic methods, orthodontic follow-up process, patient compliance.

Early orthodontic treatment of children with malocclusion requires patients and parents to fully understand the purpose and significance of early orthodontic treatment goals. Clinicians of early orthodontic treatments need to emphasize that early orthodontic treatment has a positive effect on craniofacial development, and that early orthodontic treatment of malocclusion in children is at the optimal time of growth and development. This necessitates the prevention and interception of abnormal dental and maxillofacial manifestations in children at different stages according to the genetic and environmental factors, as well as the etiological mechanisms of malocclusion. Additionally, it involves promoting coordinated and balanced dental and maxillofacial growth and development to reduce the severity and complexity of malocclusion. Early orthodontic treatment can reduce the possibility of orthognathic surgery needs for skeletal problems of patients in adult. Preoperative communication with the patient and their parents to obtain their consensus is fundamental for a successful early orthodontic treatment.

It should be emphasized that the nature of the staging and limitations of early orthodontic treatment in children, and the continuity between early orthodontic treatment and later comprehensive orthodontic treatment. The early orthodontic treatment is four-dimensional orthodontic treatment, incorporating a timeline of growth and development. It is also important to maintain a comprehensive oral health for normal dental and maxillofacial growth and development in the early orthodontic treatment for children.

Varied choices of early orthodontic treatment for children should be provided. Clinicians of early orthodontic treatments need to select the most suitable orthodontic appliance for the child based on the mechanism of child’s malocclusion, the principle of appliance design, and the patient’s compliancy to the appliance and patient’s financial ability. When multiple orthodontic methods are available to achieve the same desired effect, the orthodontist should select the method based on the patient’s and parents’ preferences, considering the effect and benefit ratio.

In addition to the expected results, the duration of treatment is also a major concern for children and their parents. Early orthodontic treatment should have a clear purpose. It is usually shorter than comprehensive orthodontic treatment. Time-consuming orthodontic treatment should be avoided in the staged treatment. In addition, since there is still time from the first phase of early orthodontic treatment to the completion of permanent dentition, efficacy of early orthodontic treatment should be emphasized. Communication before the start of treatment can allow patients and their parents to have an adequate psychological preparation.

Early orthodontic treatment requires the active cooperation of patients and their parents, and clinical compliance is an important factor in the success of early orthodontic treatment. Early orthodontic treatment focuses on problems that interfere with maxillofacial growth and development, oral health and oral functions, and the tooth alignment is not a priority to considerate, removable and functional appliances are mostly used in early orthodontic treatment. The removable orthodontic appliances require active patient cooperation to ensure accurate adaptation and secure retention otherwise the efficacy of the appliance can be compromised. In addition, early orthodontic treatment is concerned with oral function and perioral muscle activators and will also require the patient’s cooperation with appropriate oral function and perioral muscle training. The clinicians of early orthodontic treatment should also fully communicate with the patient and their parents prior to treatment regarding the need for cooperation and the lack of orthodontic effect due to poor-cooperation.

## Notes on early orthodontic treatment

### Timing of early orthodontic treatment

Early orthodontic treatment is treatment by using patient’s maxillary growth and development, correct and effective orthodontic treatment can change the abnormal dental and facial growth, and promote the coordinated development of dental and jaws. Early orthodontic treatment requires accurate timing to make a good use of growth (without delaying treatment), but also to quickly and effectively complete the staging of treatment (to avoid excessive clinical orthodontic time). For example, when addressing mandibular underdevelopment through functional treatments, initiating treatment during the pre-peak or peak of puberty growth and development can effectively correct the mandibular deficiency and reduce overall clinical treatment time.

1. The different sequence of three-dimensional growth and development of the jaws and face determines the different timing for the start of early orthodontic treatment of malocclusion on different dimensions, and clinical early orthodontic treatment should be noted.
2. The correlation between tooth eruption and occlusal development to the jaw development is week, and the timing of orthodontic treatment of dental development abnormalities is different from the correction of jaw developmental abnormalities.
3. Early orthodontic treatment is not the final treatment, and most of the early orthodontic treatments also requires later phase comprehensive orthodontic treatments in the permanent dentition. Early orthodontic treatment is mostly two-phases treatment.
4. Treatments of abnormal facial growth patterns should be postponed, and worth to wait after the completion of child’s craniofacial growth and development to make the treatment choices. Individuals with skeletal class III malocclusion have large mandibular bones. Because mandibular development could continue until late adolescence or even 20 years old, and orthodontic treatment should be postponed. Individuals with skeletal class III malocclusion with hereditary high-angle mandibular over-development (especially for male high-angle patients) often require orthodontic-orthognathic combination therapy in adulthood. Underdevelopment of the mandibular ascending branch causes front open bite (over-erupted molars). early orthodontic treatment is prone to recurrence of open bite due to incomplete vertical development of the mandible, and clinical treatment should be postponed.
5. The pursuit of early orthodontic treatment is better dental and jaw coordination and esthetics, better oral function maintenance, and more stability of treatment results. Early orthodontic treatment that does not expect to achieve better results is not necessary.

### Oral and systemic health management in children during early orthodontic treatment

Oral Health Management for Children (OHMC) refers to the comprehensive management of dental, jaw, and facial health growth and development, including early prevention, early diagnosis and early intervention, and early orthodontic treatment of malocclusion in children is the core of OHMC from embryo to adult. Since early orthodontic treatment of malocclusion in children coincides with the peak growth and development of children, the comprehensive management of children’s oral and general health cannot be neglected. It includes reasonable dietary balance, supplementation of calcium, phosphorus, and vitamins, etc.; cultivation and maintenance of good oral hygiene; timely correction of deleterious oral habits; early prevention of childhood caries, such as the use of fluoride, gutter closure, etc., to create an oral functional environment conducive to children’s health and to maintain normal growth and development of children.
